# Quantifying a Systems Map: Network Analysis of a Childhood Obesity Causal Loop Diagram

**DOI:** 10.1371/journal.pone.0165459

**Published:** 2016-10-27

**Authors:** Jaimie McGlashan, Michael Johnstone, Doug Creighton, Kayla de la Haye, Steven Allender

**Affiliations:** 1 Global Obesity Centre, Deakin University, Geelong, Australia; 2 Institute for Intelligent Systems Research and Innovation, Deakin University, Geelong, Australia; 3 Department of Preventive Medicine, University of Southern California, Los Angeles, California, United States of America; West Virginia University, UNITED STATES

## Abstract

Causal loop diagrams developed by groups capture a shared understanding of complex problems and provide a visual tool to guide interventions. This paper explores the application of network analytic methods as a new way to gain quantitative insight into the structure of an obesity causal loop diagram to inform intervention design. Identification of the structural features of causal loop diagrams is likely to provide new insights into the emergent properties of complex systems and analysing central drivers has the potential to identify leverage points. The results found the structure of the obesity causal loop diagram to resemble commonly observed empirical networks known for efficient spread of information. Known drivers of obesity were found to be the most central variables along with others unique to obesity prevention in the community. While causal loop diagrams are often specific to single communities, the analytic methods provide means to contrast and compare multiple causal loop diagrams for complex problems.

## Introduction

Complex problems can be difficult to understand and resolve due to the relationships between their multiple dynamic causes. Obesity is a prime example [1], along with other population health problems [2]. It has been suggested that any intervention seeking to tackle complexity would be better served if a shared understanding of the complexity was developed to support intervention design, implementation and evaluation [3]. Among the numerous approaches available to understand and share knowledge of complexity [4], systems science methods appear the most promising [5]. System science techniques range in their utility for community engagement and collect broad views of complexity from fully engaged, process driven methods to small group highly quantitative approaches designed primarily to generate mathematical simulation [6–8].

One technique that develops community engagement and input arises from system dynamics (SD) and particularly group model building (GMB) which creates visual grounded logic models called causal loop diagrams (CLDs) [9]. CLDs provide a method to ‘map’ the complexity of a problem of interest that comprises variables, causal relationships and polarity. *Variables* are dynamic causes or effects of the problem under study. *Causal relationships* are arrows that represent a directed cause from one variable to another. *Polarity* captures the orientation of each relationship, being either positive, where variables change in the same direction, or negative, where variables change in opposite directions.

Systems thinking and CLDs are an emerging method in public health [6], with a classic example being the Foresight obesity systems map [10]. The Foresight map brought together many of the world’s experts in obesity to develop a comprehensive picture of the factors and relationships related to obesity. The resulting ‘obesity systems map’ presents a causal diagram beginning with energy balance at an individual level and expands to a set of 108 variables that directly or indirectly influence energy balance.

To be successful, community level interventions should acknowledge the complexity of obesity by implementing multiple strategies in the community [11]. For this reason, more recent work has applied GMB techniques to develop CLDs of a complex problem from a community perspective to underpin intervention design [12]. These diagrams have been used with large numbers of community based health, government and lay people to visualize the range of, and connections between, multiple dynamic variables. The CLDs developed are an explicit representation of the shared mental model of the community group.

### Network Analysis

A CLD is naturally represented as a graph or network of relationships among a set of variables, and thus contains data that lends itself to network analysis [13]. Networks are entities comprised of nodes and edges, with edges representing relationships among nodes. Formally networks are represented as an adjacency matrix, with nodes *x*, and the presence or absence of edges between each pair of nodes *x*_*ij*_ = {0, 1}. Diverse types of problems can be represented as networks and have been a focus of scientific research. For example, social networks are networks involving interactions among social entities, such as contact, co-authorship [14] and music collaboration [15], along with other types of ‘non-social’ networks such as word co-occurrence [16], brain structure [17], yeast interaction [18] and protein networks [19]. CLDs can also be represented as a network and adjacency matrix with variables *x* and directed causal relationships between each pair of variables *x*_*ij*_.

Network analysis provides a suite of quantitative techniques that can summarise the structure of a network and quantify the importance of its elements. Understanding the structural features of a network as a whole can provide key insights into the ease or difficulty by which information, influence, or physical matter flow through the network [20]. Measures that summarize the position of nodes in a network can provide information on node importance or function in the system [17].

Network analysis has been applied to causal symptom networks to identify central symptoms for psychopathology [21] and psychiatry [22]. Post-traumatic stress disorder symptoms have also been explored via network analysis [23] along with persistent complex bereavement disorder [24], and perceived relationships between anxiety, post-traumatic stress disorder and depression [25].

In obesity, the analysis of networks has been employed in recent studies to investigate social influence on obesity and broader interdependence between social networks and obesity-related factors and outcomes including physical activity [26], food choice [27], sedentary behaviour [28], and body mass index (BMI) [29] [30]. An application to systems biology identified key biological and metabolic variables related to obesity [31]. A comprehensive map of the obesity related molecules has been recently developed and analysis of the network showed that the system’s structure resembles a scale-free network topology with well-defined variable clusters [32]. As this type of network topology is well known in network science, network theory provides useful insights into the implications of this network topology for this particular molecular system.

### Network Analysis of a Causal Loop Diagram

Quantitative network analytic techniques such as network structure summaries and centrality measures have yet to be applied to CLDs for the causes of obesity. As network analytic measures are applied in fields of network science, the application of these analytic tools allow us to leverage knowledge and gain insights into the structure and function of the CLD.

Structural metrics that summarize the entire CLD’s topology include network density, degree distribution, average path length, and modularity (described in Table 1). For example, small path lengths are often seen in ‘small-world networks’ [33] and are indicative of the network’s ability for efficient diffusion [34]. Interpretation of the network topological measures in the context of a CLD are outlined in Table 1 along with their proposed implications for intervention design.

**Table 1:** Structural network measures and their proposed interpretation for causal loop diagrams and intervention planning.

Network Analysis Measure	Definition	Interpretation in CLD	Implication for Intervention Design
**Density**	Fraction of edges present relative to the maximum possible number of edges given the set of nodes.	According to the group developing the diagram, the fraction of causal relationships that exist between pairs of variables that are identified (relative to the number of possible causal relationships, if each pair of variables was causally related).	In dense networks, change in one variable has a higher chance of causing change in other variables, and to other parts of the system. Sparse networks mean interventions likely need to ‘seed’ change in multiple parts of the network to impact the whole system.
**Degree Distribution (in and out)**	The distribution of number of edges leading to or exiting nodes in the network.	Distribution of how many causal relationships variables are involved in.	Nodes with the highest degree will act as ‘hubs’ in the network [17]. Hubs in a CLD system may be valuable for creating change interventions as they are perceived to influence or be influenced by many other variables
**Average path length**	The smallest number of ties between any two nodes in the network, on average.	Informs the interconnectedness of the CLD and its efficiency to spread change from one variable to another.	A small average path length may allow a change in one variable to cause change in others with a small amount of effort, on average.
**Modularity**	The strength of the division of node clusters in the network, which have dense inter-connections but are sparsely connected to nodes outside of the cluster [36].	Detects structural clusters in the map, which may correspond to variable themes, and measures how segregated the clusters are from each other.	If modularity is high interventions should seed change within distinct clusters and focus on variables with high betweenness centrality to facilitate the spillover of system-wide change across variables in the network.

**Fig 1:**
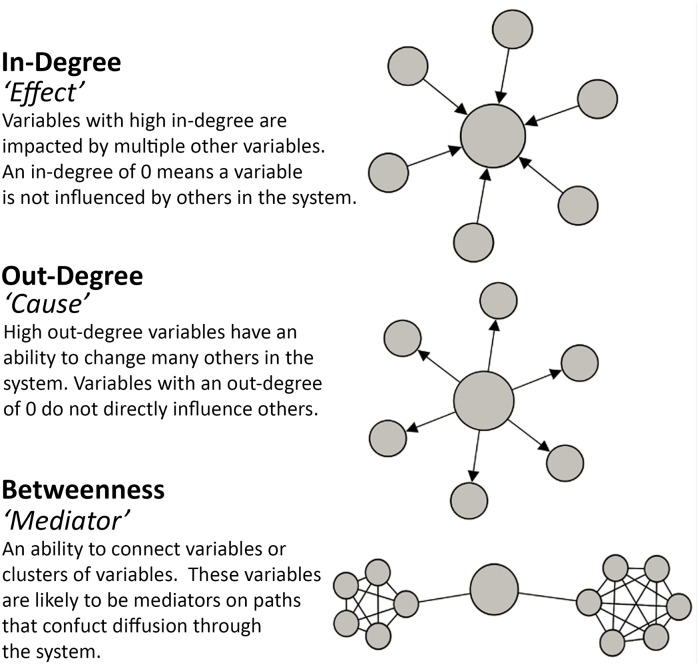
Individual node metrics: In-degree, Out-degree and Betweenness centrality interpretations for a causal loop diagram.

To gain insight into the centrality and influence of each individual node, commonly applied measures include degree and betweenness [35]. As the CLD network is directed, (not symmetric) in-degree and out-degree are considered. In-degree is the number of edges directed to a specific node, from other nodes in the network. Out-degree is the number of edges directed from a particular node, to other nodes in the network. Betweenness centrality is a measure proportional to the number of shortest paths a node lies on, with ‘shortest paths’ indicating the minimum distance (number of edges) between a pair of nodes. Nodes with high betweenness centrality lie on the paths that connect many pairs of nodes, and hence play a role in mediating or managing the flow of information between nodes in the network. Definitions of the individual node metrics and their interpretation for the CLD are outlined in Fig 1.

### Motivation

Once developed via GMB, CLDs are used in reference to the existing evidence base to develop informed approaches to systems change for obesity prevention. To date, however, it has not been clear how to determine leverage points, or the ease in which changes in one part of the system will impact other parts of the system (if at all).

The motivation of this work comes from the desire for numerical summaries of CLD structure and quantifying the importance of variables to inform intervention design.

We hypothesise that through the application of network analysis topological measures, insight will be gained about the structure of the community drivers of obesity and will allow quantitative comparisons across communities. Further, the use of common centrality measures will quantify the position and importance of variables within the system.

In this paper we seek to answer the following research question:

How does the application of network analysis to a community developed causal loop diagram advance our understanding of the system of childhood obesity drivers?

The remainder of this paper is structured as follows. The acquisition of data is outlined in the following section along with the proposed procedure for the analysis. Numerical results are presented for both the structure of the network, and the centrality of its nodes in the results section. Finally, the discussion section interprets the results in the context of obesity prevention and summarizes the future implications of this work.

## Methods

### Data

The data were originally presented by Allender *et al.* (2015) [12]. The data analysed in this submission and consent procedure for participants received ethics clearance from the institutional review board of Deakin University. Ethics Committee reference number HEAG-H 155_2014. The CLD describing childhood obesity was developed via group model building across four workshops in 2014. Data were collected with a working group of 12 participants and a final workshop with 49 members of the broader community. The working group consisted of a range of stakeholders including representatives from the Primary Care Partnership, District Health Service and Local Government.

At the completion of the workshop series, a CLD was developed depicting the drivers of childhood obesity in the community. To minimise potential bias, the CLD was constructed by iteratively seeking and implementing feedback from the working group and the broader community group. Note that the CLD analysed here was developed one workshop later than that presented in [12].

### Network Analysis Procedure

The CLD developed describing childhood obesity was represented as a directed un-weighted network to allow the application of network analysis.

Structural network measures (Table 1) were used to quantitatively summarize characteristics of the network as a whole. This, in combination with network theories and network science, provide insight into the qualities and function of this system: for example, it’s stability, efficiency in spreading information or change, and other characteristics likely to be relevant to planning system-wide intervention and change.

Individual node summaries such as in-degree, out-degree and betweenness centrality are described in Fig 1. Centrality measures inform the role and importance of obesity drivers in the diagram. The structural measures and centrality results can provide quantitative summaries that could be used to inform the design of effective interventions.

Analysis and visualisation were conducted using Gephi [37], which applies well-established algorithms for computing network statistics. The algorithm for calculating shortest paths is provided by Brandes (2001) [38].

## Results

### Structure of the Causal Loop Diagram

**Fig 2:**
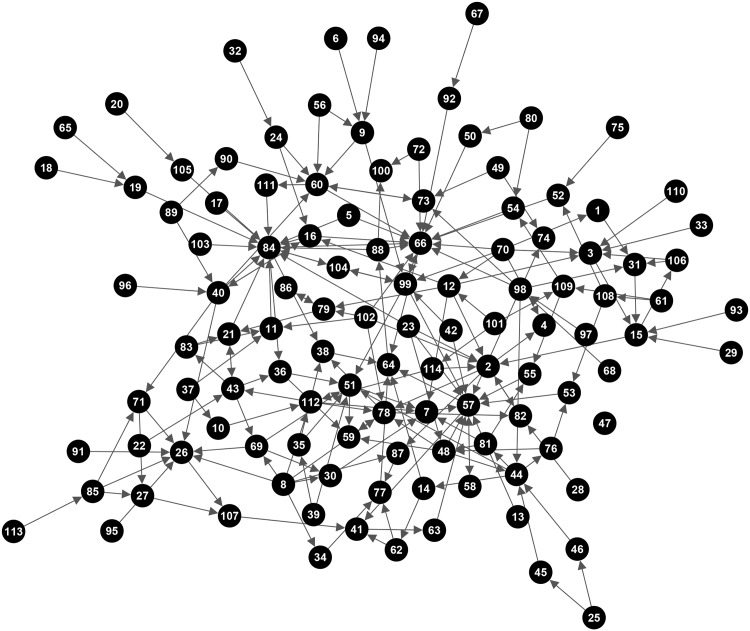
Community developed CLD of obesity drivers displayed as a directed network.

The causal loop diagram for childhood obesity drivers developed by the participant group is shown in Fig 2. The CLD comprises 114 variables and 209 relationships, with one node *(variable 47)* with no edges (an isolate). Table 2 shows the global network structure summaries. S1 Table provides a key for node ID.

**Table 2:** Summary of network statistics for the CLD.

Nodes	Edges	Density	Av. Path Length	Modularity
114	209	0.016	4.65	0.56

**Fig 3:**
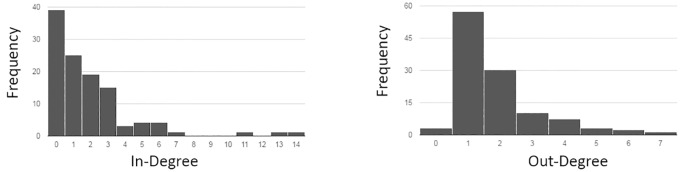
Distribution of node in and out degree (number of in and out bound edges for each node) for the community developed obesity CLD.

The network **density** is 0·016, meaning that this network contains 1·6% of the possible edges expected in a completely interconnected network. **Degree distributions** are shown in Fig 3. The distribution of node in-degree ranges from 0 to 14, and shows a large number of nodes have low in-degree, with few larger *hubs* present. The distribution of node out-degree ranged from 0 to 7 and similar to in-degree is *heavy-tailed*. Unlike in-degree, however, it is rare for nodes to have a an out-degree of 0, meaning its unlikely for a variable to not have an impact on any others.

The **average path length** in the network is 4.65 meaning variables are able to reach each other by following 4.65 causal paths, on average.

The two variables that are most distant, ‘Water taste’ and ‘Overeating’ (113 → 77) have a shortest path of 16, meaning the CLD has a *diameter* of 16.

The network **modularity** of 0.56, calculated via Gephi’s modularity function [36], indicates the presence of structural clusters of variables in the network.

### Variable Centrality

**Fig 4:**
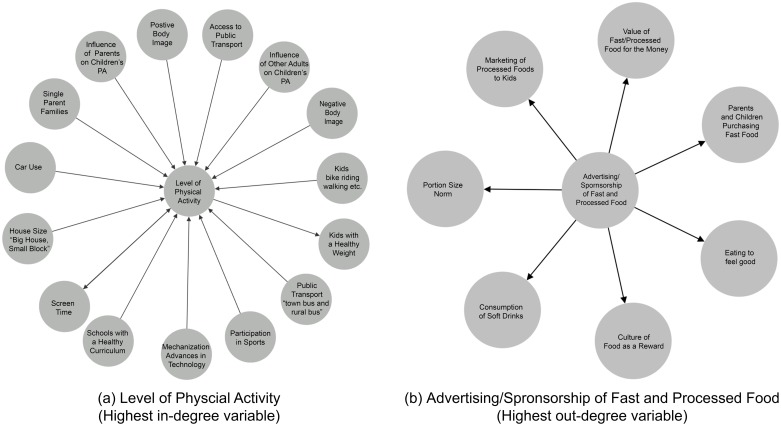
A summary of the relationships to and from the variables with the highest in-degree and out-degree in the system, respectively.

Fig 4a shows the variables associated (as a cause or effect) with the variable with the highest **in-degree**. ‘Level of Physical Activity’ *(variable 66)* is effected by 14 other variables in the system (in-degree = 14). Following this, is ‘Participation in Sports’ *(84)* (in-degree = 13), ‘Junk Food Consumption’ *(57)* (in-degree = 11) and ‘Consumption of Soft Drink’ *(26)* (in-degree = 7).

As shown in Fig 4b, ‘Advertising/Sponsorship of Fast and Processed Food’ *(variable 8)* has the ability to influence 7 other variables in the CLD and has the maximum **out-degree**. ‘Schools with a Healthy Curriculum’ *(98)* has an out-degree of 6, followed by ‘Available Time’ *(12)*, ‘Fear and Risk Averse Society’ *(40)* and ‘Single Parent Families’ *(102)* directly causing 5 factors in the system (out-degree = 5).

**Fig 5:**
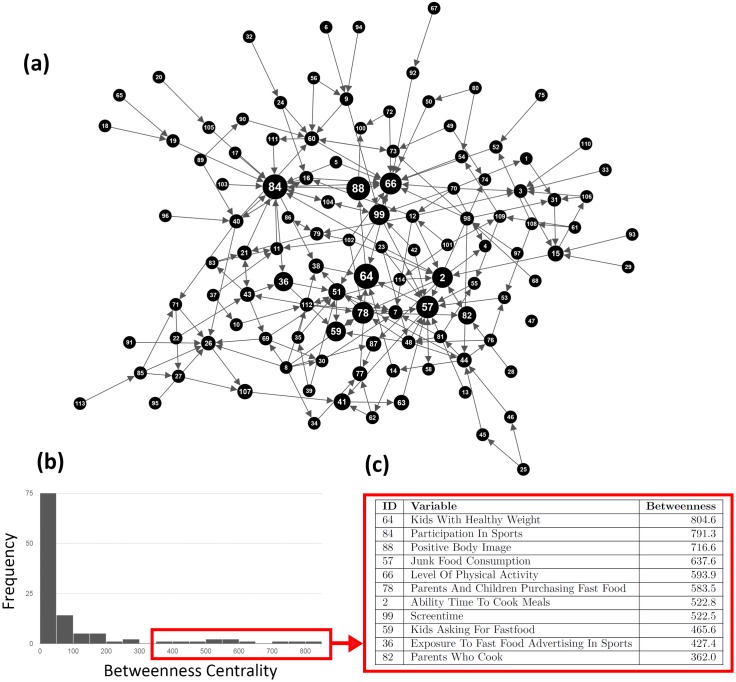
Variables with the highest betweenness centrality- the ‘mediators’ of the causal loop diagram shown (a) by node size in the network, (b) the distribution of values and (c) a table of values for nodes with the highest betweenness centrality.

Variables with a high **betweenness centrality** and the distribution of values are shown in Fig 5. Similar to the distribution of degree, the majority of the variables have a low betweenness with a small number of high value outliers. ‘Kids with Healthy Weight’ *(variable 64)* ranks highest (betweenness = 804.6), followed by ‘Participation in Sports’ *(84)* (betweenness = 791.3), ‘Positive Body Image’ *(88)* (betweenness = 716.6), and ‘Junk Food Consumption’ *(57)* (betweenness = 637.6).

## Discussion

### Statement of Principle Findings

The results have implications for understanding of the topological structure and key drivers of childhood obesity, specifically within (and perhaps not limited to) this community.

A number of the topological measures computed for the CLD were similar to that of well studied, real networks, providing confidence in the representation of the CLD as a directed network and the application of standard network analytic methods.

For example, the pairing of low-density and low average path length is characteristic of ‘small-world’ network topologies [39]. While heavily tailed degree distributions (Fig 3) are characteristic of ‘scale-free networks’ [40]. Small world and scale free networks, which are observed in many empirical networks (social networks, the world wide web, and biological networks), have properties that are well known to influence the function and resilience of the network, and provide useful insights into the function of the CLD.

The low density means the network is sparse, and changes in one variable may not impact other parts of the system as quickly as a dense network. Thus, planning leverage points will have to be more strategic and acknowledge the topology of the network. Low density could also imply sophisticated development of the CLD, as although all factors are indirectly related (via childhood obesity), only the direct, proximal causal relationships were identified by the participant group.

Measuring path length gives further insight by evaluating distances of minimum causal chains between variables. According to Hovmand (2013) [9], long causal chains are often disregarded due to the likelihood of interference. The maximum shortest path (diameter) of length 16, from ‘Water Taste’ to ‘Overeating’, could be an example of this, and it may not be wise to place confidence in this chain. However, on average, the path length between variables in the diagram is 4.65 meaning the CLD resembles a network with a structure efficient in the spread of information and influence [41].

The modularity value of 0.56 is indicative of a divided network [42], highlighting the presence of variable clusters in the CLD. This result further asserts the importance of acknowledging variables with high betweenness centrality (Fig 5), as their ‘mediating’ role will facilitate the spillover of change from one cluster to other clusters in the system.

If an intervention focused around the consumption of soft drink, for example, were to eventually impact ‘Level of Physical Activity’, it would need to traverse the high betweenness variables ‘Kids with a Healthy Weight’, ‘Positive Body Image’ and ‘Junk Food Consumption’. An intervention around town infrastructure, may eventually influence the consumption of junk-food via ‘Kids with a Healthy Weight’ and ‘Screen Time’.

Many variables with high betweenness were identified in multiple feedback loops during the development of the CLD [12], which further asserts their power as a leverage point [43]. For example, the variable ‘Positive Body Image’ is a part of a feedback loop. ‘Positive Body Image’ directly influences ‘Participation in Sports’ and ‘Level of Physical Activity’. ‘Participation in Sports’ impacts ‘Kids with a Healthy Weight’, which in turn reinforces improvement in ‘Positive Body Image’.

High in-degree variables, those that are most causally influenced by others in the system, are indeed well recognised drivers of childhood obesity, including those related to physical activity [44] along with the consumption of both unhealthy food and sugar sweetened beverages [10].

The advertising of unhealthy food and curriculum of local schools have the greatest influence, (highest out-degree), meaning they have the ability to influence the greatest number of other variables in the system and may be powerful in initiating system-wide change. Often, high out-degree variables had an in-degree of zero (‘Advertising/Sponsorship of Fast and Processed Food’, ‘Mechanization Advances in Technology’, ‘Single Parent Families’ and ‘[Local Sporting] Club size’). This means that although these variables may be influence change in many other variables, the community has not identified variables that cause change to these important influencer variables and therefore it is possible that the community may not have an ability to alter them. ‘School with Healthy Curriculum’, however, impacts many others (out-degree = 6), can be changed by ‘School Canteen Policy’, which could be a promising leverage point that will affect variables such as ‘Healthy Literacy’, ‘Junk Food Consumption’ and ‘Normalising Healthy Culture’.

### Strengths and weaknesses of the study

Network analysis provides a novel way to quantify drivers of obesity in a community led CLD. The CLD was developed using a method that ensures strong comparability and repeatability between sessions. This work extends on other attempts to quantify networks by expressly focusing on grounded community perspectives of drivers of disease. The centrality analyses in this study did not consider polarity and delay of the relationships, which will be considered in a subsequent study. It is also noted that results presented in this paper are specific to a single community’s systems map, however, the network analysis interpretations are applicable to all causal loop diagrams.

### Strengths and weaknesses in relation to other studies

Some of the key drivers identified in the analysis are well studied causes of obesity and are present in the Foresight map [10], but others are specific to the community. This is due to the difference in development of the two diagrams. The CLD in this work was developed by community participants rather than experts, to ensure the diagram was of us for intervention design specific to the community. Finegood *et al.* (2010) [1] also applied network analysis techniques to an existing systems map of obesity drivers, however, the objectives and methods in this project focus on numerical rather than visual summaries.

Similar work has been conducted ‘beneath the skin’ by Jagannadham *et al.* (2016) [32] who found the structure of the biological obesity system has a heavy-tailed degree distribution and high modularity. Above the skin, we have noticed a similar scale-free structure in the community drivers of obesity.

### Implications of the study

We found that the community developed CLD of obesity drivers studied in this paper has a structure similar to other well studied networks. With this knowledge, conclusions regarding the structure of a problem can be extracted.

For population health problems, the insight of central variables can aid intervention planning by understanding their role in the system. Global network measures will provide insight into how the system’s structure can be leveraged for more efficient system-wide change.

Computing quantitative measures also allows comparison among CLDs. A comparison of diagram structure and central variables before and after an intervention could be insightful to measure changes in the problem over time. Spatial comparisons, such as comparing the structure CLDs between communities, could allow towns with similar results to leverage successful interventions.

The framework presented in this paper may provide the means to gain insight into causal loop diagrams, not just for obesity, but for all complex problems.

### Unanswered questions and future research

A comparison with additional CLDs for obesity, along with other problems could give interesting insight into the similarities or differences of the topological structure found in this research. Acknowledgement of some similarities between this map and the Foresight map have been made, though further insight or quantification of their overlap using network analysis techniques could provide valuable information.

An alternative method to quantify the contents of systems maps for decision making and intervention design are stock and flow diagrams [45], which have been applied to population health problems [46] [47]. Conversion of CLDs to stock and flow models, however, relies on assumptions and development of mathematical equations for elements in the model. The quantitative measures used in this research allow application directly to a CLD immediately following its creation. Future work could consider the results from obtained network analysis to inform the quantification and simulation of system dynamics models. For example, by simulating changes that target the most central nodes in the CLD.

System dynamic models should not considered as static, and should change as the problem evolves. Therefore, the CLD and results presented in this study should not be taken as a ‘final’ model [12]. A promising method to allow real-time collection of system data in the community is wireless sensor networks [48], which can allow for monitoring and recognition of activity and changing states of the system [49]. Such an approach may provide a means to update information about the system and models in real-time [50], and thus deploy more adaptive and timely intervention strategies [51].

## Conclusion

The main contribution of this paper is the application of network analysis to a well grounded community developed causal loop diagram of obesity drivers. This method is a novel way to identify central variables in a systems map for obesity and to gain an in-depth understanding of the structure of the diagram and thus of the problem. The CLD network of community obesity drivers is sparse, and has characteristics observed in other empirical networks known to be efficient for information distribution. Centrality analysis was applied to all variables to identify their role in the system. Insight from network analysis can aid community groups in intervention design by considering a variable’s position in the network. Well known causes of obesity ranked highest in this study increasing confidence in the proposed method, though interesting insights unique to this community were also uncovered.

## Supporting Information

S1 Table Variable ID key. Variable names corresponding to ID.
(CSV)

S2 Table Causal loop diagram network data. Edge list of the connections in the community developed causal loop diagram for childhood obesity.
(CSV)
